# Relative telomere length and senescence-associated inflammatory cytokines as blood-based prognostic markers in patients with advanced or resectable gastro-oesophageal adenocarcinoma

**DOI:** 10.1038/s41416-025-03221-z

**Published:** 2025-11-17

**Authors:** Alan E. Bilsland, Eilidh McCulloch, Sofie Degerman, Mattias Landfors, Jon Wadsley, Lucy Wall, Catherine Thompson, Iva Damyanova, Leslie Samuel, Russell Petty, Ankit Jain, Liz-Anne Lewsley, Antonia MacMillan, Martin MacLeod, Jennifer Walker, Carol McCormick, Elaine McCartney, Patricia Roxburgh, Jamie Stobo, Fiona Thomson, T. R. Jeffry Evans, W. Nicol Keith

**Affiliations:** 1 School of Cancer Sciences, University of Glasgow, Glasgow, UK; 2 Department of Medical Biosciences, and of Clinical Microbiology, Umea University, Umea, Sweden; 3 Weston Park Cancer Centre, Sheffield, UK; 4 Edinburgh Cancer Centre, Western General Hospital, Edinburgh, UK; 5 University Hospitals Morecambe Bay NHS Trust, Morecambe Bay, UK; 6 Mid Yorkshire NHS Trust, Pinderfields Hospital, Wakefield, UK; 7 Aberdeen Royal Infirmary, Aberdeen, UK; 8 University of Dundee, Dundee, UK; 9 Russells Hall Hospital, Dudley, UK; 10 Glasgow Oncology Clinical Trials Unit, School of Cancer Sciences, University of Glasgow, Glasgow, UK; 11 NHS Greater Glasgow & Clyde, Glasgow, UK

**Keywords:** Gastric cancer, Cancer

## Abstract

**Background:**

Combination chemotherapy provides significant survival advantage in patients with advanced gastro-oesophageal adenocarcinoma compared with best supportive care. Peri-operative chemotherapy is standard of care for patients with operable disease. We hypothesised that biomarkers of genomic instability and inflammation may have clinical utility in these patients.

**Methods:**

We initiated open-label, non-randomised biomarker studies in patients with advanced disease due to receive Epirubicin, Cisplatin and Capecitabine (ECX)/Epirubicin, Cisplatin and Fluorouracil (ECF) or Epirubicin, Oxaliplatin and Capecitabine (EOX)/Epirubicin, Oxaliplatin and Fluorouracil (EOF) regimens (advanced study, *n* = 375), and in patients planned to receive perioperative chemotherapy with the same regimen (peri-operative study, *n* = 306). Relative telomere length (RTL) in peripheral blood mononuclear cells (PBMCs) and plasma levels of 10 inflammatory cytokines were analysed to determine association with progression-free and overall survival, and response. Blood samples were collected prior to treatment and on each treatment cycle. Both studies comprised biomarker discovery and validation cohorts. Here we report analysis of the discovery cohorts.

**Results:**

In advanced disease, high pre-treatment levels of IL8 and IL10 associated with poor Progression Free Survival (PFS) and Overall Survival (OS) in univariate analysis, and IL6 with poor OS. In multivariate analysis, IL6 and IL8 remained associated with OS, and IL8 with PFS. In the perioperative study, cytokine levels were significantly lower and no relationships were observed. There was no association between RTL and any endpoint in either study.

**Conclusions:**

Pre-treatment RTL was not prognostic, although IL6/IL8 were negative prognostic factors in advanced disease. Levels of these were lower in patients with localised disease, suggesting an association with disease progression. Further analysis of systemic inflammatory status in gastro-oesophageal adenocarcinoma may be promising for development of future predictive biomarker signatures.

## Introduction

Gastro-oesophageal adenocarcinoma has a poor prognosis, accounting for 10,000 deaths per year in the UK. Combination chemotherapy results in a significant survival advantage in patients with advanced gastric cancer when compared with best supportive care in randomised clinical trials [1–3]. High response rates may be obtained in these tumours by the use of protracted venous infusional 5FU, epirubicin and cisplatin—the ECF regimen [4] and a significantly improved response rate (45%) and median survival (8.9 months), with significantly less toxicity, compared to the FAMTX regimen [5].

Subsequently, capecitabine was shown to be equivalent to infusional 5-Fluorouracil (ECX) and the combination of epirubicin, oxaliplatin, and capecitabine (EOX) gave a further improvement in overall survival [6] such that these regimens were standard of care in the UK at the time that this study was initiated. Nevertheless, the response rates with these regimens, and the overall survival, remain disappointing with significant toxicity in patients who do not respond to treatment. Therefore, the development of biomarkers to aid patient selection for treatment would be advantageous for this patient population.

Peri-operative chemotherapy is the standard of care in the UK for patients with operable gastric or gastro-oesophageal junction adenocarcinoma and the ECF or ECX regimens were used at the time that this study was initiated [7, 8]. It is not yet possible to identify sub-sets of patients who will benefit from peri-operative chemotherapy, versus those patients in whom there is no benefit but the potential for considerable toxicity. Similarly, it is not yet possible to identify sub-sets of patients with chemotherapy-resistant disease at the time of surgical resection and in whom further (post-operative) chemotherapy is not likely to be beneficial. Therefore, biomarkers to aid patient selection for peri-operative chemotherapy, particularly the post-operative component of this treatment regimen, would be advantageous for this patient population also.

Telomere shortening, genomic instability and inflammatory signalling may be key events in the initiation and progression of gastro-oesophageal cancer [9]. We previously reported that low and high grade Barret’s oesophagus exhibits high levels of senescence-associated β-galactosidase staining, with reduced levels in gastric and oesophageal adenocarcinoma [10]. Thus, aging and senescence mechanisms may be involved in disease progression. However, senescence is a complex phenotype, involving multiple overlapping hallmarks of aging and cancer including metabolic remodelling, stable epigenetic alterations, and inflammatory signalling [11].

Telomere length in peripheral blood has been investigated as a potential biomarker in a wide range of health conditions. Short telomeres in peripheral blood are associated with the incidence of both GI and head and neck cancers [12–15], although there is conflicting evidence for a relationship between peripheral blood telomere length and the risk of progression to oesophageal adenocarcinoma [16, 17]. We previously reported that increased plasma levels of the DNA damage associated secreted senescence marker N-Acetylglucosaminidase (NAG) is an independent prognostic indicator of poor overall survival in advanced gastro-oesophageal adenocarcinoma in a retrospective analysis [18]. NAG is upregulated and secreted by aging cells with shortened telomeres and, like peripheral blood telomere length, is associated with organismal aging [19]. In subgroup analysis, we found that increased NAG following initiation of fluoropryimidine/platinum therapy was positively associated with treatment response, though this was based on a small sample size [18]. Together these results suggest that telomere length and secreted senescence markers could be useful in monitoring progression and possibly also response in gastro-oesophageal cancer.

Consequently, we initiated two multi-centre studies (UK Clinical Research Network studies 12434 and 12435), in which we prospectively analysed relative telomere length (RTL) in peripheral blood at baseline (pre-chemotherapy) and subsequent chemotherapy cycles in advanced gastro-oesophageal patients receiving ECF/ECX or EOF/EOX, and in patients eligible to receive perioperative chemotherapy with the same regimens [20, 21]. Additionally, we analysed plasma levels of 10 inflammatory cytokines involved in the senescence-associated secretory response (SASP) [22]. A major aim of both studies is the longitudinal analysis of these markers and these studies are ongoing. Here we report first results from our baseline (pre-chemotherapy) analysis.

## Patients and methods

### Patients

Two separate, open, non-randomised studies were performed. Both trial protocols are available at protocols.io (10.17504/protocols.io.n92ldznenv5b/v1; 10.17504/protocols.io.ca5csg2w). For the advanced disease study [21], eligible patients were those with inoperable disease comprising either histologically or cytologically confirmed locally advanced or metastatic gastric, gastro-oesophageal junction, or oesophageal adenocarcinoma who were candidates for palliative therapy and due to start 1st-line chemotherapy with either the ECX/ECF (epirubicin, cisplatin and capecitabine/5-FU) or EOX/EOF (epirubicin, oxaliplatin and capecitabine/5-FU) regimens, and who had at least one uni-dimensionally measurable lesion. Additionally, patients randomised in the NCRI REAL-3 study to receive EOX + panitumumab were also eligible.

For the peri-operative study [20], eligible patients were those with histologically or cytologically confirmed gastric or gastro-oesophageal junction (including Type 1 lower oesophageal) adenocarcinoma, who were considered to have operable disease as determined by a Multi-Disciplinary Team (MDT) and who were candidates for peri-operative chemotherapy with either the ECF/EOF or ECX/EOX regimens. Additionally, patients randomised in the NCRI STO3 study to receive ECX + bevacizumab were also eligible.

For both studies, patients were aged ≥18 years, with an estimated life expectancy of ≥3 months, and had received no systemic anti-cancer chemotherapy or radiotherapy within the previous 6 weeks. Patients’ pre-treatment evaluation, treatment and assessments during treatment, were unaffected by participation in this study and were the standard management for these patients.

### Blood sampling and processing

20 ml of blood were collected prior to initiation of palliative or pre-operative chemotherapy (baseline) and before each subsequent chemotherapy cycle into K2E EDTA vacutainers (BD Biosciences, Oxford, UK).

After completion of palliative chemotherapy in patients with advanced disease, blood samples were collected where possible at each follow-up visit until documented disease progression. In the perioperative study, further samples were collected on completion of pre-operative chemotherapy, prior to post-operative chemotherapy, on day 1 of each cycle of post-operative chemotherapy, and at the time of any subsequent documented disease recurrence. Our longitudinal design is intended to determine whether changes in any candidate marker during the course of therapy have predictive value, although here we report baseline analysis.

Plasma and PBMC samples were processed at participating sites and stored within 3 h of collection. For plasma preparation, whole blood was centrifuged for 10 min at 1500 *g*. Supernatant was transferred to a clean tube and centrifuged again for 10 min at 1500 g prior to aliquoting and freezing. For PBMC isolation, 5 ml whole blood was layered over 5 ml histopaque-1077 hybri-max (Sigma, Gillingham, UK) and centrifuged at room temperature (20 min, 800 × *g*). The plasma layer was discarded and the PBMC layer was removed to a fresh tube and diluted in 10 ml ice-cold PBS. This was centrifuged (10 min, 4 °C, 500 × *g*) to pellet the PBMCs. Supernatant was removed and the pellet was resuspended in 1 ml of ice-cold PBS and pelleted again (5 min, 4 °C, 500 × g). Supernatant was removed and the pellet was stored at −70 °C.

### Relative telomere length (RTL) quantitative PCR

DNA was extracted from PBMCs on the BioRobot® M48 workstation (Qiagen; Hilden, Germany), using MagAttract DNA Blood midi kits (Qiagen) according to the manufacturer’s protocol. DNA quality and quantity were determined by spectrophotometry using the Nanodrop instrument (Thermo Scientific, Wilmington, USA). RTL was determined using qPCR as described in [23]. Each sample of DNA was analysed in triplicate wells in separate Telomere (TEL) and Haemoglobin subunit beta (HBB) (single-copy gene) qPCR reactions on the ABI7900HT instrument (Applied Biosystems, Waltham, Massachusetts, USA).

Each 20 μL TEL/HBB reaction contained 17.5 ng DNA, 1X PCR buffer II (Applied biosystems), 1.7(TEL)/2.5(HBB) mM MgCl_2_, 2.5(TEL)/5(HBB) mM DTT, 0.2 mM dNTP, 150 nM ROX, 0.2X SYBRgreen, 1.25 U AmpliTaq gold (Applied biosystems), and 0.1/0.9 μM forward/reverse TEL primers or 0.4 μM forward/reverse HBB primers [23]. The TEL/HBB cycling conditions were 95 °C 10 min, 25 cycles at 95 °C 15 s, 56 °C 60 s (TEL) or 40 cycles at 95 °C 15 s, 54 °C 60 s (HBB). A reference sample (CCRF-CEM cell line) DNA was included in all runs to determine the samples’ relative telomere length (RTL). Relative telomere length (RTL) was calculated by the formula: sample DNA 2^−ΔCt (average CtTEL- average CtHBB)^/ Reference (CCRF-CEM cell line) DNA 2^−ΔCt (average CtTEL- average CtHBB)^.

### MesoScale Discovery (MSD) electro-chemiluminescent cytokine immunoassays

Cytokine assays were performed using MesoScale Discovery 10-plex pro-inflammatory human cytokine panel 1 kits, according to the manufacturer’s instructions (#K15049G-2, MSD, Rockville, MD). 25 µl of plasma was diluted 1:2 in diluent 2 and incubated at room temperature with capture antibodies pre-coupled to the assay plates for 2 h on a plate shaker at 700 rpm. Wells were washed three times with 150 µl of 1 x wash buffer and 25 µl of detection antibody mix added to each well. Detection antibodies were IFN-γ, IL-1β, IL-2, IL-4, IL-6, IL-8, IL-10, IL-12p70, IL-13 and TNF-α. Each was provided at 50 x concentration and diluted to 1 x for detection in a single mastermix using diluent 3. After further 2 h’ incubation at room temperature on a plate shaker at 700 rpm, each well was washed three times with 150 µl of 1 x wash buffer.

Finally, 150 µl of read reagent was added to each well and luminescence was quantified immediately on a QuickPlex-SQ120 plate reader (MSD, Rockville, MD). Quantification was performed using MSD proprietary software, interpolating the kit-specific calibrator blend which was added to each plate as a 7-point, 4-fold serial dilution containing a standard curve for each analyte. Reactions were performed in duplicate, and each plate contained high, medium and low controls for each analyte. All patient samples were analysed on two independent occasions.

### Statistical hypotheses and methods

For the advanced disease study, the primary endpoint was the association of baseline telomere length with clinical outcome. Sample size calculations for RTL analyses targeted 80% power assuming equal-sized groups split by median RTL at baseline. However, our analyses were continuous, using the method of fractional polynomials (MFP) to determine appropriate transformations [24, 25]. Outcome assumptions in the advanced disease study were based on an overall response rate of 45%, median progression-free survival (PFS) of 6.5 months, and a median overall survival (OS) of 10 months with ECX/EOX [6] We specified at least 25% difference between high/low RTL groups for prognostic interest (32.5% vs 57.5%). For both PFS/OS in advanced disease we targeted a Hazard Ratio (HR) between high/low RTL groups of at least 2 for prognostic interest, that is, median PFS of 4.35 vs 8.65 months and median OS of 6.65 vs 13.35 months.

For the perioperative chemotherapy study, we assumed a 3-year relapse-free survival (RFS) of 40% and 3-year OS of 45% with the ECF regimen [7]. Again, we specified a HR of 2 between high/low RTL groups (27.5% vs 52.5% for RFS; 32.7% v 57.3% for OS).

To detect these magnitudes of difference with 80% power at the 1.7% 2-sided level of statistical significance in the advanced disease study (adjusted for three co-primary endpoints) required 155 patients for objective response and 120 and 130 patients for PFS and OS respectively. In the perioperative study, the 2-sided significance level was 2.5% (adjusted for two co-primary endpoints) and required 144 patients for both RFS and OS. The sample size for the advanced study targeted the largest number required for any comparison (155 patients).

To detect RTL as an independent prognostic factor we increased sample sizes by 6.25%, allowing for modest correlation (0.25) between RTL and other prognostic factors, leading to sample sizes of 165 and 153 for the advanced disease and perioperative studies respectively. To allow independent validation of prognostic models developed using exploratory biomarkers in the initial cohorts, we also aimed to recruit subsequent validation cohorts of the same size to each study. Therefore, the total target sample sizes were 330 and 306 patients. Here we report results obtained for analysis of RTL and a panel of inflammatory cytokines in the first cohorts of each study.

For the advanced disease study, objective response was determined from the patient’s standard of care radiology reports but measurements by RECIST1.1 were not mandated nor was there external independent review of the radiology images. PFS (advanced disease study) is defined as the time from registration to disease progression or death, whichever occurs sooner. However, disease assessments by CT scanning was performed as clinically indicated on completion of palliative chemotherapy, rather than by regular interval imaging. RFS (peri-operative chemotherapy study) is defined as the time from registration to relapsed disease or death, whichever occurs sooner. For disease recurrence to occur, a patient must have completed surgery. These patients who progressed/died prior to surgery or had surgery abandoned had event times set to 0 to avoid overestimating RFS.

In both studies, OS is defined as the time from registration to death (all causes). Date of death for individual patients was determined from patient records either during hospital follow-up or from patient records if they had been discharged from regular attendance at the investigator site.

## Results

### Baseline RTL is not associated with overall survival

Patient characteristics are shown Table 1. At the times of data cut-off (12/10/2015 for the advanced study and 28/02/2021 for perioperative study), the median durations of follow-up were 25.1 months (advanced disease) and 60.1 months (perioperative study). At the time of analysis, 145 patients in the advanced disease study had progressed, with 123 patients deceased. In the perioperative study, 87 of the patients with potentially curative disease treated with peri-operative chemotherapy had deceased.

**Table 1:** Patient characteristics.

Age	All patients (perioperative study, *n* = 165)	Completed surgery (*n* = 128)	All patients (advanced study, *n* = 182)
Median	63	64	64
Range	27–85	27–85	26–86
Gender			
Female	26	20	45
Male	139	108	137
Ethnicity			
Caucasian	163	126	179
Black	0	0	0
Asian	1	1	1
Other	1	1	2
Primary tumour site			
Stomach	53	48	54
Oesophagus	60	44	92
GO-Junction	52	36	36
Differentiation			
Well	5	7	9
Moderate	65	46	49
Poor	75	59	103
Unknown	19	5	21
Missing	1	11	0
T-stage			
0	0	4	0
1	3	14	7
2	32	21	7
3	105	72	94
4	13	8	45
X	12	5	23
Missing	0	4	6
N-stage			
0	70	65	17
1	70	22	79
2	13	24	49
3	7	14	18
X	5	0	17
Missing	0	3	2
M-stage			
0	156	75	32
1	0	3	141
X	8	44	9
Missing	1	6	0
Evaluable			
RTL	147	114	163
Cytokines	165	128	182

Patients with T-stage 0 had no detectable primary tumour. As shown in the table, these patients were exclusively in the perioperative group after pre-operative chemotherapy; that is, these patients had achieved a pathological complete response in the primary tumour after pre-operative chemotherapy. No patients with advanced disease or in the perioperative group at baseline had T stage 0.

Baseline (pre-treatment) RTL in PBMCs of patients in the advanced disease (*n* = 163) and peri-operative chemotherapy (*n* = 147) studies was inversely correlated with age (Supplementary Fig. 1). In the perioperative patient group there was a trend toward longer baseline RTL in PBMCs of patients with T1/T2 tumours versus T3/T4 and Tx, but there were no other associations with TNM stage (supplementary Fig. 2) or pathological grade or primary tumour site (Supplementary Figs. 3 and 4). Female patients had higher mean baseline RTL than male patients in the perioperative study but not in the advanced study (supplementary fig. 5).

**Fig. 1:**
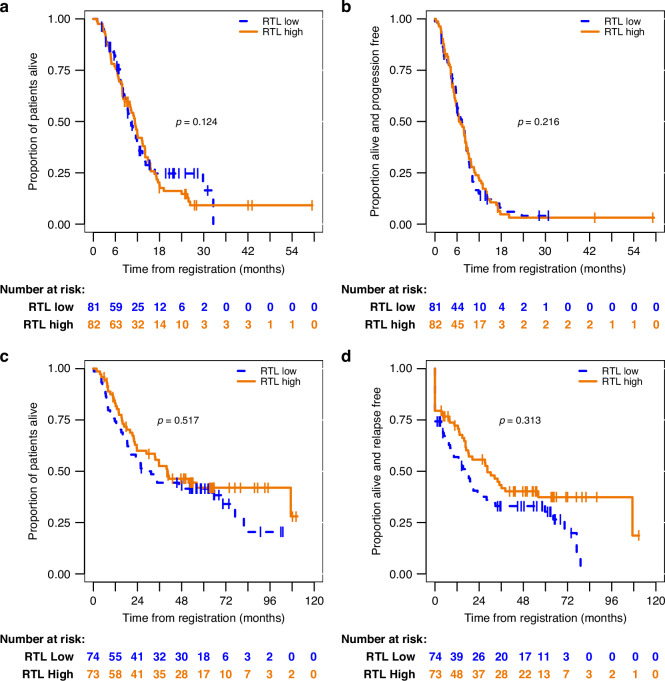
RTL is not associated with survival in gastro-oesophageal cancer in advanced or perioperative settings. Patients were stratified by RTL levels in each case (above/below median levels). Kaplan–Meier plots are shown for illustrative purposes only. Data were analysed continuously using the method of fractional polynomials. **a** OS in the advanced study. 40 patients were censored at the date they were last known to be alive. **b** PFS in the advanced study. 18 patients were censored. **c** OS in the perioperative study.60 patients were censored. **d** RFS in the perioperative study. 52 patients were censored.

In patients with advanced disease, there was no significant relationship between OS and baseline RTL levels by univariate Cox regression analysis at the 2-sided 1.7% level (Fig. 1a, Table 2). Similarly, there was no significant difference in PFS between these groups (Fig. 1b, Table 2).

**Table 2:** Univariate survival and response regression analysis results for the advanced and perioperative studies.

Endpoint	*N* (events)	*B*	SE	*p*-value	exp(B) (exp(B) for 1 SD increase)	95% CI for exp(B) (1 SD increase)	
						Lower	Upper
Advanced study							
PFS	163 (145)	−0.738	0.597	0.216	0.478 (0.895)	0.148 (0.751)	1.540 (1.067)
OS	163 (123)	−0.967	0.628	0.124	0.380 (0.865)	0.111 (0.719)	1.302 (1.040)
ORR	163	1.974	1.144	0.0845	7.200 (1.345)	0.764 (0.961)	67.815 (1.882)
Perioperative study							
RFS	147 (95)	−0.619	0.613	0.313	0.539 (0.906)	0.162 (0.747)	1.791 (1.098)
OS	147 (87)	−0.434	0.669	0.517	0.648 (0.933)	0.175 (0.756)	2.406 (1.151)

Cox regression analysis results are shown for baseline RTL versus relationship with OS/PFS in the advanced study and with PFS/RFS in the perioperative study. Univariate logistic regression results are additionally shown for baseline RTL relationship with ORR in the advanced study. All analyses used the method of fractional polynomials. However, no transformation of the RTL variable was selected in any analysis. B – regression parameter estimate; SE – estimated standard error of regression parameter estimate. For OS, PFS and RFS, exp(B) is the hazard ratio for a one unit increase. For ORR, exp(B) is the odds ratio for a one unit increase. Also shown in brackets are estimates for exp(B) and confidence intervals for a 1 standard deviation increase in baseline RTL level.

Similar findings were observed for OS and RFS in the perioperative study (Fig. 1c, d, and Table 2). Although the Kaplan–Meier plot for RFS does appear to suggest a survival advantage in the high RTL group, the relationship did not reach statistical significance in Cox regression at the 2-sided 2.5% level (Table 2, *p* = 0.313).

Since the univariate analyses of survival endpoints did not reach statistical significance, multivariate analyses were not performed. However, sensitivity analyses were undertaken to account for potentially confounding effects of age. Additionally, gender was included as a variable in sensitivity analysis of the perioperative study results due the observed association between RTL and gender in that study, as noted above (Supplementary Fig. 5). Linear models were fit for the baseline RTL data, with age and/or gender as the explanatory variables. The residuals from these models were then extracted and used as the covariates in a Cox model. The interpretation is that positive/negative residual value indicates the patient has a larger/smaller RTL value respectively than expected given their age or age and gender.

For OS analyses, in the perioperative study a likelihood ratio test comparing the null linear model (adjusted for age only) and a more complex model (adjusted for age and gender), indicated that gender significantly improved the model fit (*p* = 0.0099). Therefore, the final model was adjusted for both age and gender. In advanced disease, addition of gender did not significantly improve the model relative to age alone (*p* = 0.359). There was no correlation between any survival endpoint in either study and whether baseline RTL value is higher/lower than expected for given age or age and gender (Kaplan–Meier plots of OS using these adjusted models are shown in Supplementary Fig. S6). Therefore, RTL in PBMCs at baseline in patients treated with platinum/fluoropyrimidine therapy appears not to be a prognostic indicator either in advanced disease or in the perioperative setting.

### IL6, IL8, and IL10 are negatively associated with survival in advanced disease

Our study protocol aimed to interrogate a range of other markers known to be involved in cellular senescence. Specifically, we also aimed to investigate markers of the senescence associated secretory response which we have previously found to be modulated by candidate druggable senescence targets [22]. We analysed expression of a panel of 10 inflammatory cytokines (IFNγ, IL1β, IL2, IL4, IL6, IL8, IL10, IL12, IL13, and TNFα) in baseline plasma from 182 and 165 patients from the primary cohorts of the advanced and perioperative studies. Multivariate survival analyses were performed only if the univariate analysis reached statistical significance at the 2-sided 10% level, after adjustment for multiple testing using the Benjamini-Hochberg false-discovery rate approach [26]. These studies were exploratory and did not form part of our main statistical hypothesis.

Of the 10 cytokines analysed, 5 (IL1β, IL2, IL4, IL12, IL13) were frequently below the assay range. These were assigned the categorical status of “detected” if at least one of the assay replicates in both repeats was in range. Otherwise, these were assigned as not detected (“categorical cytokines”). In the advanced disease study, the proportion of patients with detectable IL1β was significantly higher in gastro-oesophageal junction cancers than in other sites (*p* = 0.006) and IL12 was detected more frequently in gastric cancers, although this was borderline significant (*p* = 0.051). There were no other associations with clinico-pathology features among the categorical cytokines and none were associated with either survival endpoint or with response (Supplementary Table S1). However, several cytokines were detectable only in a very small number of patients.

The other cytokines (IFNγ, IL6, IL8, IL10, TNFα) were within the assay range and were analysed continuously. Baseline IFNγ did not reach significance for either PFS/OS in advanced disease (Supplementary Table 1). For TNFα, the relation to both OS/PFS was highly sensitive to a single patient outlier. After removal of this patient from the analysis, neither endpoint showed significant association with TNFα levels (see Supplementary Table 1 and legend text).

**Fig. 2:**
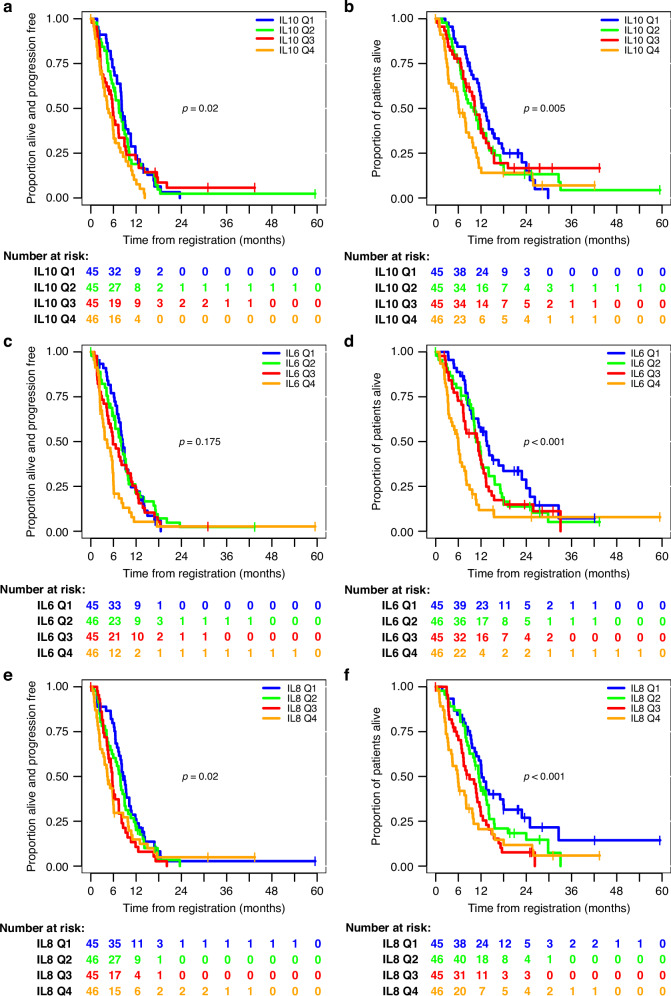
Univariate associations of IL10, IL6, and IL8 with survival in advanced disease. Kaplan–Meier plots are shown for illustrative purposes only. Data were analysed continuously using the method of fractional polynomials (Table 3). Relations are shown between **a** PFS and **b** OS for IL10; **c** PFS and **d** OS for IL6; **e** PFS and **f** OS for IL8. Patients were stratified by quartile of cytokine levels.

Univariate analyses of IL10 levels in the advanced disease patients study suggested an inverse association with survival (Fig. 2 and Table 3). In interpretation of the hazard ratios, note that the method of fractional polynomials selected an inverse transformation for both OS/PFS (Table 3). For PFS (Fig. 2a), the hazard ratio for IL10^−0.5^ was 0.708 (90% CI: 0.582–0.860, FDR-adjusted *p* = 0.020). For OS (Fig. 2b), the hazard ratio for IL10^−1^ was 0.890 (90% CI: 0.836–0.947, FDR-adjusted *p* = 0.005).

**Table 3:** Univariate Cox regression analysis results for OS/PFS in the advanced study.

Endpoint (MFP transformation)	*N* (events)	*B*	SE	p-value (FDR adjusted)	exp B	90% CI for exp B	
						lower	Upper
Advanced study							
PFS (IL10^−0.5^)	181 (165)	−0.346	0.119	0.020	0.708	0.582	0.860
OS (IL10^^−1^)	181 (147)	−0.117	0.038	0.005	0.890	0.836	0.947
PFS (IL6/10)	182 (166)	0.280	0.134	0.175	1.323	1.061	1.649
OS (Log(IL6/10))	182 (148)	0.401	0.088	<0.001	1.494	1.293	1.726
PFS (IL8/10)	182 (166)	0.085	0.022	0.020	1.088	1.049	1.129
OS (Log(IL8/10))	182 (148)	0.398	0.089	<0.001	1.488	1.286	1.722

Cytokines deemed significant for one or both endpoints are included. Analysis results for all cytokines considered are given in supplementary table 1. All analyses used the method of fractional polynomials (MFP) and transformations of the variables selected by the algorithm are shown. B – regression parameter estimate; SE – estimated standard error of regression parameter estimate. Exp(B) is the hazard ratio for a one unit increase. Also shown in brackets are estimates for exp(B) and confidence intervals for a 1 standard deviation increase in transformed baseline level of cytokines.

High IL6 levels also corresponded with poor survival in advanced disease (Fig. 2c, d, and Table 3), although this did not reach significance at the 5% level for PFS after FDR adjustment (HR = 1.323, 90% CI: 1.061–1.649, FDR-adjusted *p* = 0.175). For OS, the hazard ratio was 1.494 (90% CI: 1.293–1.726, FDR-adjusted *p* < 0.001. Similar results were also obtained for univariate analysis of IL8 levels (Fig. 2e, f, and Table 3) with a hazard ratio for PFS of 1.088 (90% CI: 1.049–1.129, FDR-adjusted *p* = 0.020) and 1.488 (90% CI: 1.286–1.722, FDR-adjusted *p* < 0.001) for OS.

Gender, TNM stage, and tumour site were considered along with IL10 for the multivariate PFS/OS models in advanced disease (Table 4). Backwards elimination variable selection retained T stage/gender in the PFS model and gender only in the OS model. In both cases, IL10 was not transformed or rescaled. The IL10 term in the PFS model, but not the OS model, remained significant (PFS model, *p* = 0.039; OS model, *p* = 0.103. Thus, IL10 may be an independent prognostic indicator of poor PFS but not OS in advanced disease.

**Table 4:** Multivariate Cox regression analysis results for OS/PFS in the advanced study.

Variable (MFP transformation)	*B*	SE	p-value	exp B	90% CI for exp B	
					lower	Upper
IL10 multivariate PFS model (*N* = 152, events = 138)						
IL10 (N.A.)	0.043	0.021	0.039	1.044	1.009	1.081
T-stage; T4 (N.A.)	-0.505	0.199	0.011	0.604	0.435	0.837
Gender; male (N.A.)	0.453	0.211	0.031	1.573	1.113	2.224
IL10 multivariate OS model (*N* = 152, events = 120)						
IL10 (N.A.)	0.039	0.024	0.103	1.040	1.000	1.081
Gender; male (N.A.)	0.382	0.200	0.0565	1.465	1.055	2.036
IL6 multivariate OS model (*N* = 182, events = 148)						
IL6 (Log(IL6/10))	0.438	0.090	<0.001	1.550	1.337	1.797
Gender; male (N.A.)	0.444	0.197	0.025	1.559	1.127	2.156
IL8 multivariate PFS model (*N* = 182, events = 166)						
IL8 (IL8/10)	0.084	0.022	<0.001	1.087	1.048	1.128
Site; stomach (N.A.)	−0.279	0.183	0.126	0.756	0.560	1.021
Site; OGJ (N.A.)	−0.137	0.207	0.507	0.872	0.620	1.226
IL8 multivariate OS model (*N* = 182, events = 148)						
IL8 (IL8/10)	0.093	0.021	<0.001	1.097	1.061	1.135
Gender; male (N.A.)	0.355	0.194	0.067	1.427	1.037	1.964

Analyses were performed for those cytokines deemed to have significant univariate association with survival. Note that for IL10, 29 patients were excluded from analysis due to unevaluable T-stage. All analyses used the method of fractional polynomials and selected variable transformations are shown where relevant. Exp(B) is the hazard ratio for a one unit increase. Also shown in brackets are estimates for exp(B) and confidence intervals for a 1 standard deviation increase in transformed baseline level of cytokines.

*N.A*. not applicable, variable was not transformed, *B* regression parameter estimate, *SE* estimated standard error of regression parameter estimate.

Since IL6 did not reach statistical significance in univariate analysis of PFS, multivariate analysis was not performed. In OS analysis, variable selection retained only gender. IL6 was scaled and log-transformed (Table 4), and remained associated with poor overall survival. The hazard ratio for log(IL6/10) was 1.550 (90% CI: 1.337–1.797, *p* < 0.001).

In multivariate analysis of PFS for IL8, variable selection retained tumour site (Table 4). IL8 was again rescaled (IL8/10). and the scaled IL8 term was highly significant in the multivariate model (HR = 1.087, 90% CI = 1.048–1.128 *p* < 0.001). In multivariate analysis of OS, gender was retained and the IL8 term was rescaled as above. Scaled IL8 remained significantly associated with poor survival (HR = 1.097, 90% CI: 1.061–1.135, *p* < 0.001). Therefore, IL8 appears to be an independent predictor of poor PFS/OS in advanced disease.

**Fig. 3:**
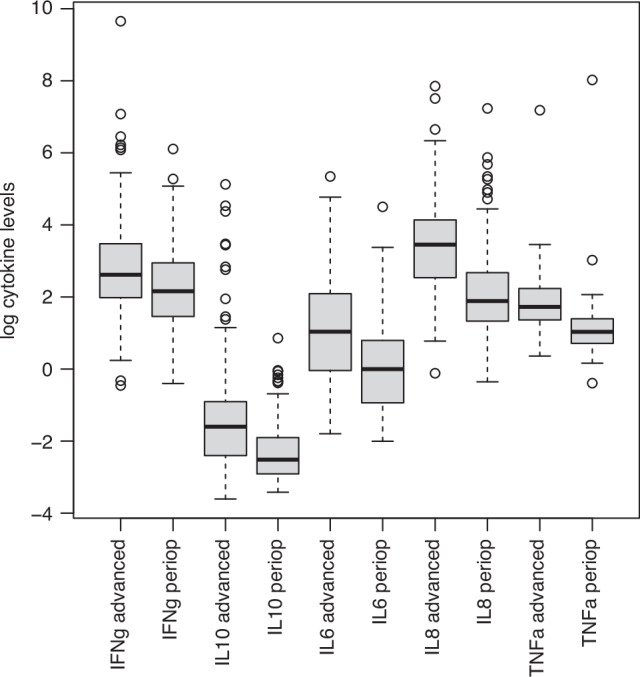
Increased inflammatory profile in advanced disease. Distributions of measured log(pg/ml) concentrations of each cytokine in all patients on both studies are shown.

No significant associations were observed for either RFS or OS in the perioperative study for any of the cytokines analysed here (not shown). Notably, however, levels of each cytokine that could be analysed continuously were significantly lower in the periperative patients than in patients with advanced disease (Fig. 3): in each case, the 75th percentile of plasma concentrations measured in perioperative patients was below the median observed in advanced disease. These results may suggest that progression from localised to advanced disease in gastro-oesophageal adenocarcinoma is associated with an increasingly pro-inflammatory profile of plasma cytokines.

## Discussion

Short telomeres in peripheral blood have been reported as a risk factor for gastric cancer [13, 15]. During recruitment to this study, two reports suggested that telomere length in PBMCs/leukocytes is associated with survival in gastric cancer [27, 28]. Our main finding here is that relative telomere length in peripheral blood prior to therapy is not a prognostic marker in gastro-oesophageal adenocarcinoma patients treated with ECF/ECX or EOF/EOX either in palliative or perioperative settings. There are important methodological differences between our study and those in [27, 28]. In the study by Qu and colleagues [27], patients did not receive chemotherapy treatment prior to surgery. While this is not explicitly stated in the study of Tahara and colleagues [28], the authors do discriminate surgery and chemotherapy groups, so it is likely these patients also did not receive prior therapy.

Therefore, differences between our conclusions may simply reflect the treatment regimens. We did not find significant relations between primary site and RTL; nor was primary site a significant variable in any multivariate analysis in the cytokine study. Beyond the differences in treatment, is also plausible that differences between our studies may also partly reflect methodological differences in measurement of telomeres and/or the heterogeneity of peripheral blood cell populations. For example, Pepper and colleagues recently argued against the application of mean telomere length measurements in blood in relation to cancer outcomes due to the intrinsic heterogeneity [29].

We also demonstrate that IL6 and IL8 are inversely associated with overall survival in advanced but not localised disease. In multivariate analysis, IL10 was also significantly associated with poor PFS but not OS. Although 29 patients were excluded from the IL10 analysis, 120/138 events were analysed for OS/PFS, which was sufficient for the target power in the RTL part of the study. However, all cytokine results are exploratory, since analyses of these markers was not specified in the original study plan. However, if similar observations are made in our validation cohorts, confidence would increase that these are robust prognostic markers in the advanced disease setting. Interestingly, patients treated in the perioperative setting also had substantially lower levels for all cytokines that could be quantitatively analysed here. It is an interesting possibility that accelerating cytokine levels might have value in predicting progression. We are currently investigating these markers longitudinally in both studies, and using orthogonal methods. These results will be reported elsewhere.

The current difficulty in predicting and optimising response to treatment in gastro-oesophageal adenocarcinoma is unsurprising given heterogeneity in the molecular features of the disease [30, 31]. However, chronic inflammation may also underlie the pathology of the disease [32]. In particular, the microsatellite instability (MSI) subtype, which is characterised by increased tumour mutational load and neo-antigen expression, and the EBV subtype are associated with high numbers of tumour infiltrating lymphocytes [33]. Both IL6/IL8 play myriad roles in the tumour microenvironment, regulating processes such as cell growth, survival, angiogenesis, epithelial-mesenchymal transition, and polarisation of infiltrating immune cells.

IL-6 promotes direct activation of JAK/STAT signalling and cross-talk with other growth-factor regulated pathways [34]. In the tumour microenvironment, it is produced by multiple cells including cancer cells, cancer-associated fibroblasts, and immune cells [35]. In combination with other cytokines it plays a role in polarisation of Th2-cells and also induces Th17 differentiation [36]. Systemically, plasma IL-6 is also linked to cachexia [37]. IL-8 is produced by macrophages, endothelial cells and epithelial cells, as well as tumour cells, and promotes infiltration of neutrophils and myeloid-derived suppressor cells into the tumour microenvironment [38]. Like tumour-associated macrophages, neutrophils in the tumour microenvironment can also be polarised to by various factors to pro-tumourigenic, immunosuppressive N2 neutrophils which themselves secrete high levels of IL-8 and may induce further recruitment [39].

To our knowledge, this is the first study to compare circulating levels of these cytokines in connection with prognosis in both advanced and perioperative settings in adequately powered and primarily Caucasian populations, although poorer patient outcomes have been associated with their elevated levels in several other studies. A retrospective Taiwanese study in operable gastric adenocarcinoma patients found IL-6 to be an independent prognostic factor for OS, although patients were treated between 1999 and 2002, prior to the widespread adoption of perioperative chemotherapy [40]. In contrast, in a Korean study of gastric carcinoma patients who underwent gastrectomy, elevated serum IL-6 was significantly associated poorer disease status and OS in univariate but not multivariate analysis although it is unclear whether these patients received perioperative treatment [41]. Two other smaller studies also linked serum IL-6 and survival in operable gastric cancer without perioperative treatment [42, 43]. The relationship between circulating IL-8 and prognosis appears to be understudied in gastric cancer [44]. However, a number of immunohistochemistry studies have been performed in gastric cancer and a recent metanalysis concluded that high expression is negatively correlated with OS [45].

Improved understanding of immunological phenotypes of gastro-oesophageal cancer is of major current importance since, recently, immune checkpoint inhibitors (ICIs) have shown promise in some patients [46]. PD-L1 combined positive score (CPS), which calculates the ratio of immunohistochemically PD-L1 positive tumour cells, lymphocytes and macrophages to viable tumour cells, has emerged as a key stratification biomarker for ICI therapy in oesphagogastric patients [47, 48]. However, there are still a proportion of patients with high CPS who may not benefit from PD-1 inhibitor therapies and may experience unnecessary adverse events, while some patients with lower CPS may benefit though would not be eligible. Further investigation of the immune and inflammatory profiles in the TME and also in circulation will be required to expand knowledge in this complex area. It is plausible that cytokine measurements could help predict advanced cancer patient response to ICI therapies and related adverse events [49]. Indeed, multiple cytokines regulate expression of the PD-L1/PD-1 axis [50].

In summary, we believe that further investigation of pro-inflammatory signatures may have value in identifying response predictors for platinum/fluoropyrimidine therapy gastro-oesophageal cancer. A major aim of both studies is to evaluate the markers studied longitudinally. It is, for example, plausible that accelerating cytokine levels or rapid RTL decrease between early treatment cycles may be useful variables in a response model. Beyond our ongoing longitudinal studies of the markers identified here, we are also currently extending these findings in the advanced study with more detailed plasma profiling using a 92 immune oncology protein assay. Furthermore, we are investigating inflammatory markers and immune cell populations, including CPS-scoring, in the TME of biopsy material from patients in the advanced study to potentially provide an indication of the relationship between systemic inflammation, the TME and tumour progression.

## Supplementary information

Supporting information

## Data Availability

The clinical data for both studies is held on the University of Glasgow servers within the Glasgow Oncology CTU. Laboratory data for both studies is held on the University of Glasgow servers within the Translational Pharmacology & immunology Laboratory. The RTL studies Investigators are committed to furthering cancer research by sharing of de-identified individual patient data from the studies with others in the field, who wish to access these data for high quality peer-reviewed research. The Trial Management Group (TMG) can consider proposals from other researchers and will commit to sharing individual patient level data to the maximum extent subject to individual study constraints related to 1) ethical approval and informed consent; 2) contractual and legal obligations, including data sharing agreements; 3) publication timelines (data will not usually be shared prior to publication of primary results). All proposals will be reviewed for scientific merit by the TMG. Only data relevant to the objectives of a particular proposal will be provided. To discuss the potential sharing of the RTL studies’ related data and for proposal submission to the TMG, please contact Professor Jeff Evans (j.evans@crukscotlandinstitute.ac.uk).
