# Correction: Relative telomere length and senescence-associated inflammatory cytokines as blood-based prognostic markers in patients with advanced or resectable gastro-oesophageal adenocarcinoma

**DOI:** 10.1038/s41416-025-03298-6

**Published:** 2025-12-19

**Authors:** Alan E. Bilsland, Eilidh McCulloch, Sofie Degerman, Mattias Landfors, Jon Wadsley, Lucy Wall, Catherine Thompson, Iva Damyanova, Leslie Samuel, Russell Petty, Ankit Jain, Liz-Anne Lewsley, Antonia MacMillan, Martin MacLeod, Jennifer Walker, Carol McCormick, Elaine McCartney, Patricia Roxburgh, Jamie Stobo, Fiona Thomson, T. R. Jeffry Evans, W. Nicol Keith

**Affiliations:** 1 School of Cancer Sciences, University of Glasgow, Glasgow, UK; 2 Department of Medical Biosciences, and of Clinical Microbiology, Umea University, Umea, Sweden; 3 Weston Park Cancer Centre, Sheffield, UK; 4 Edinburgh Cancer Centre, Western General Hospital, Edinburgh, UK; 5 University Hospitals Morecambe Bay NHS Trust, Morecambe Bay, UK; 6 Mid Yorkshire NHS Trust, Pinderfields Hospital, Wakefield, UK; 7 Aberdeen Royal Infirmary, Aberdeen, UK; 8 University of Dundee, Dundee, UK; 9 Russells Hall Hospital, Dudley, UK; 10 Glasgow Oncology Clinical Trials Unit, School of Cancer Sciences, University of Glasgow, Glasgow, UK; 11 NHS Greater Glasgow & Clyde, Glasgow, UK

**Keywords:** Gastric cancer, Cancer

Correction to: *British Journal of Cancer* 10.1038/s41416-025-03221-z, published online 17 November 2025

In this article, the author’s name W. Nicol Keith was incorrectly written as Nicol W. Keith.

The original article has been corrected.

