# Supplementary material for: Relative telomere length and senescence-associated inflammatory cytokines as blood-based prognostic markers in patients with advanced or resectable gastro-oesophageal adenocarcinoma
Source: Br J Cancer. 2025 Nov 17;134(2):208–17. doi: 10.1038/s41416-025-03221-z (PMC12820329; doi:10.1038/s41416-025-03221-z)
Supplement: Supplementary file 1 — Supporting information [file 41416_2025_3221_MOESM1_ESM.docx]

A


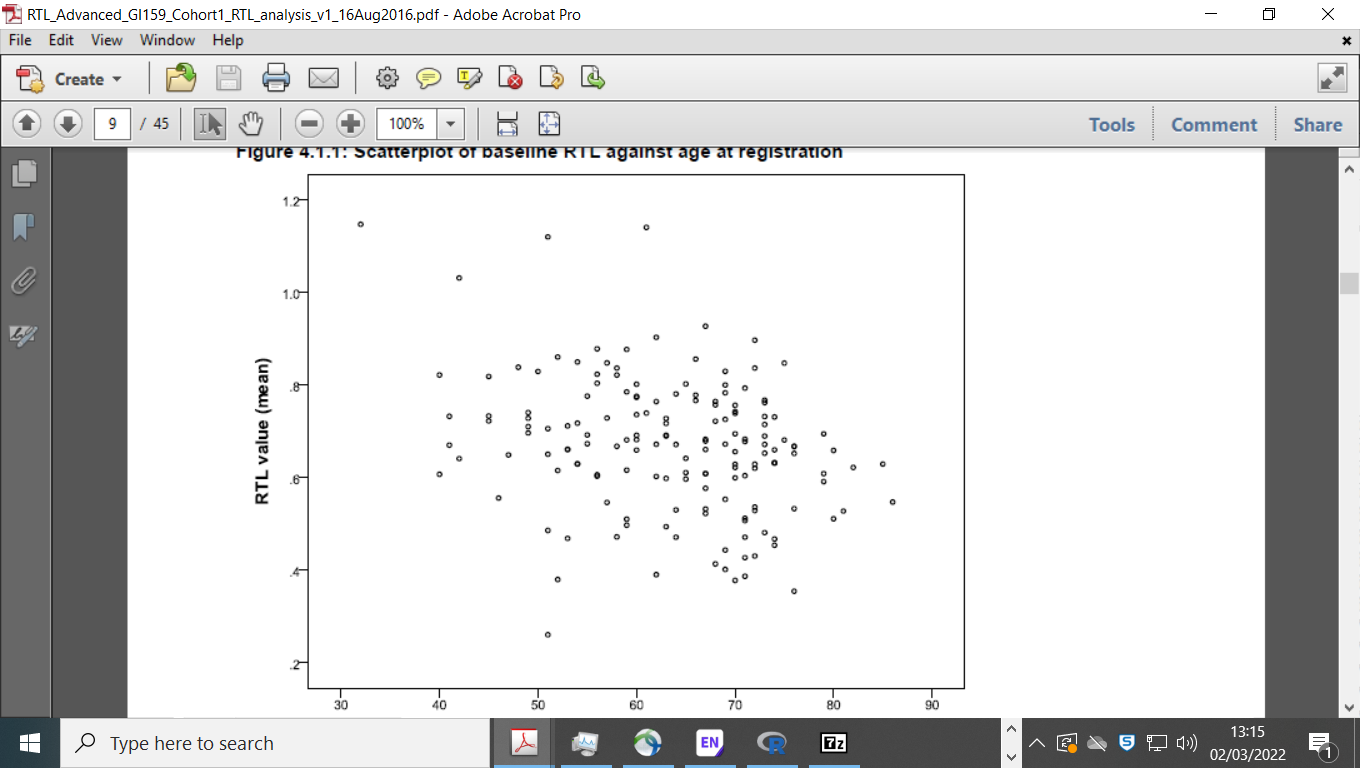


B


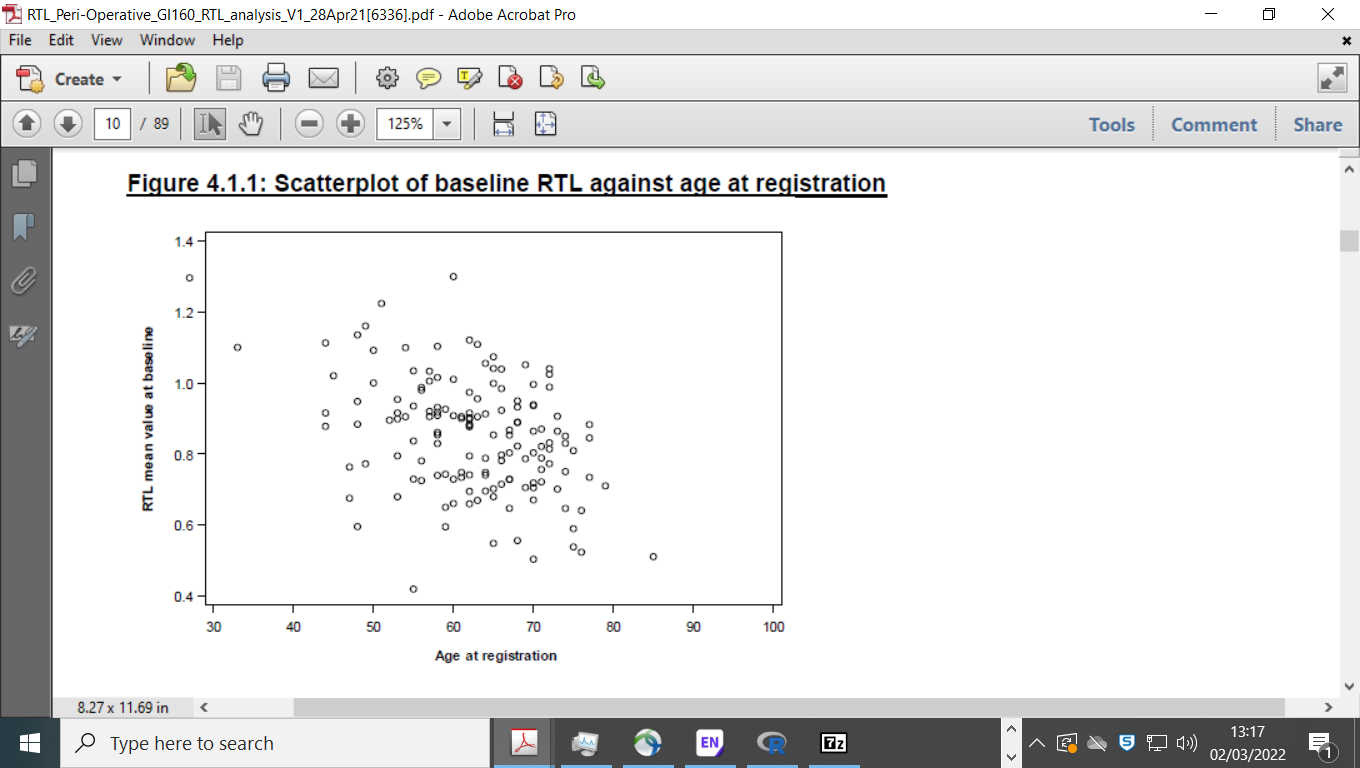


**Supporting figure S1: Relative telomere length is negatively associated with age at registration.** Pearson correlation suggests a modest negative correlation between RTL and age in (A) advanced study (ρ = -0.29, p < 0.001) and (B) perioperative study (ρ = -0.39, p < 0.001).

**
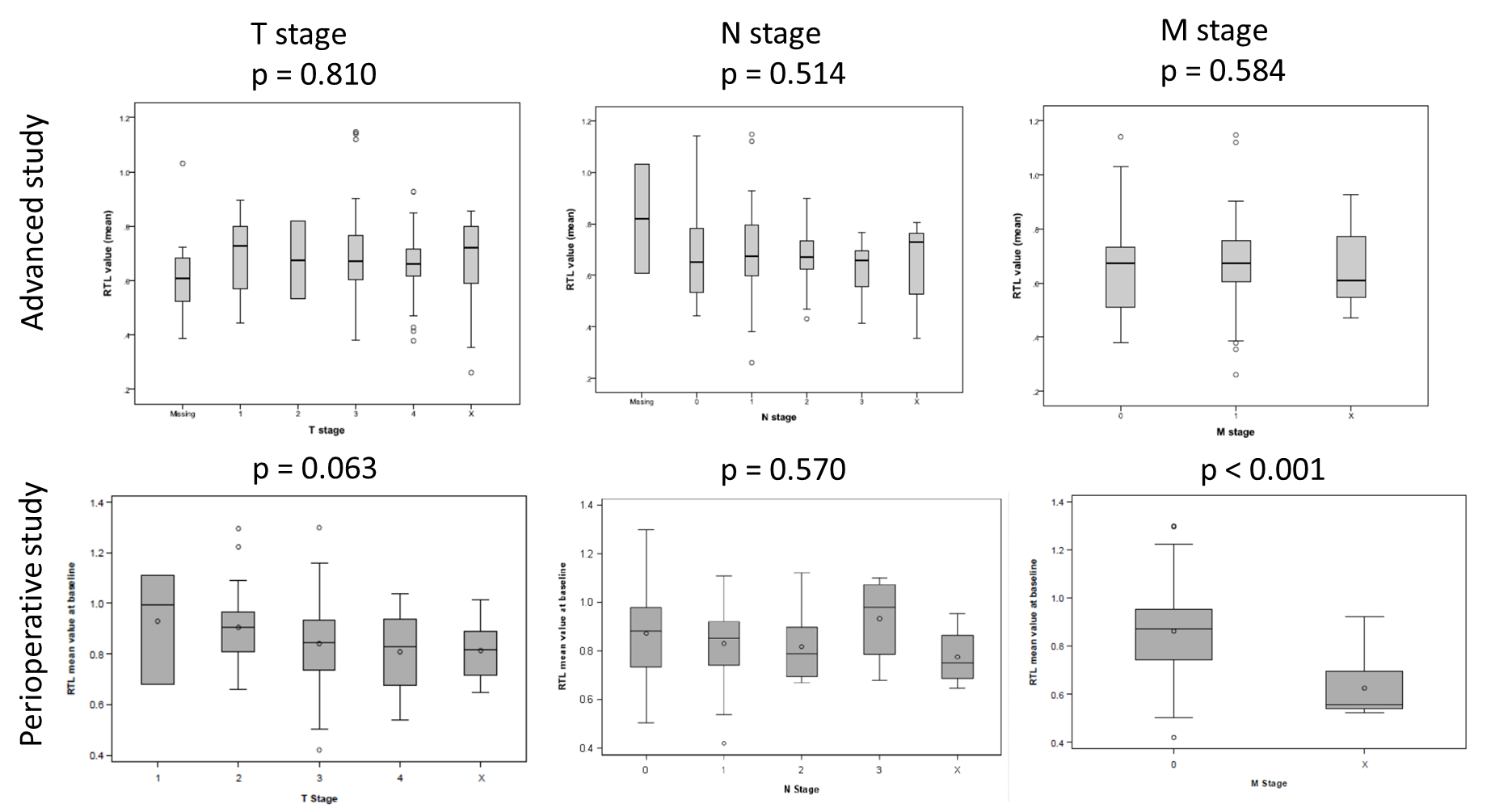
**

**Supporting figure S2: No significant association between RTL and TNM stage.** Top panels, advanced disease; lower panels, perioperative. Comparisons of baseline RTL and T, N, and M stage were performed by Mann-Whitney or Kruskal-Wallis test, as appropriate (p values shown in figure). Since patients in the perioperative study did not have metastasis, M-stage analysis was not performed.

**
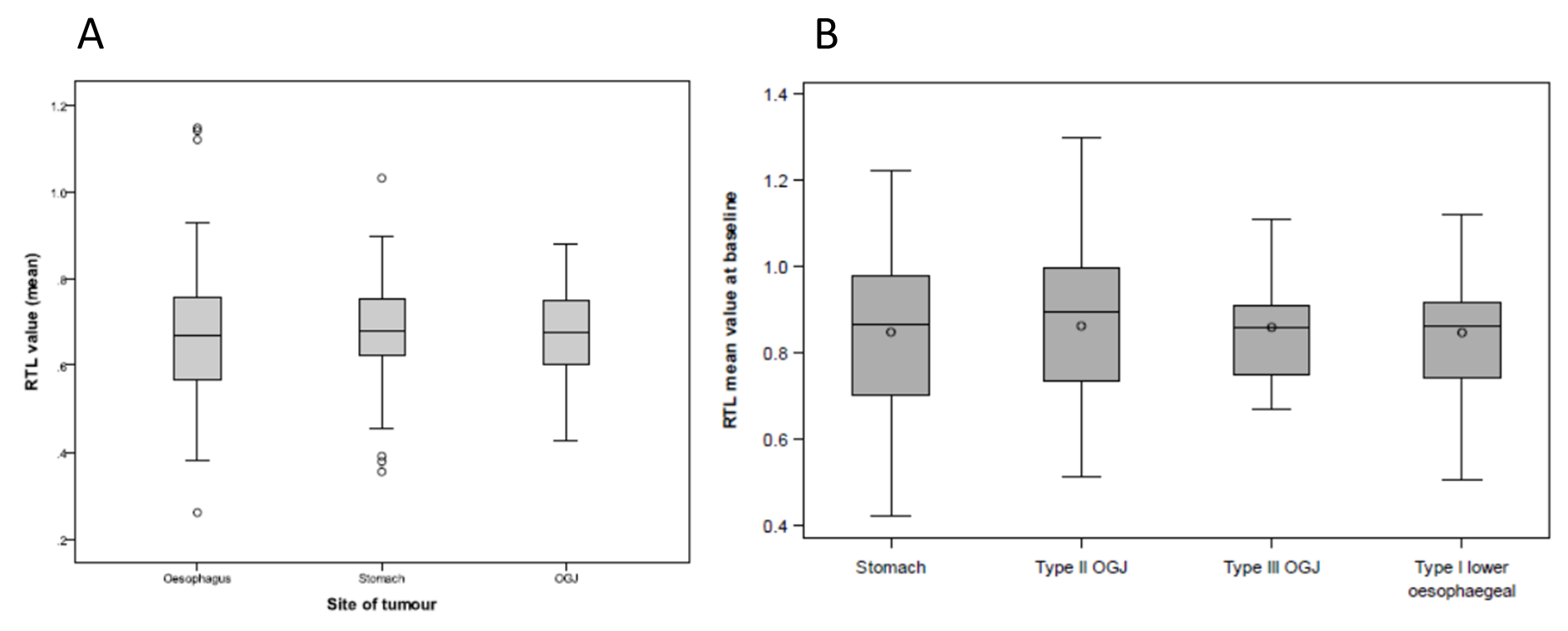
**

**Supporting figure S3: No significant association between baseline RTL and primary site.** (A), advanced disease; (B), perioperative disease. Comparisons were made by Kruskal-Wallis test (p = 0.757, advanced disease; p = 0.983, perioperative).

**
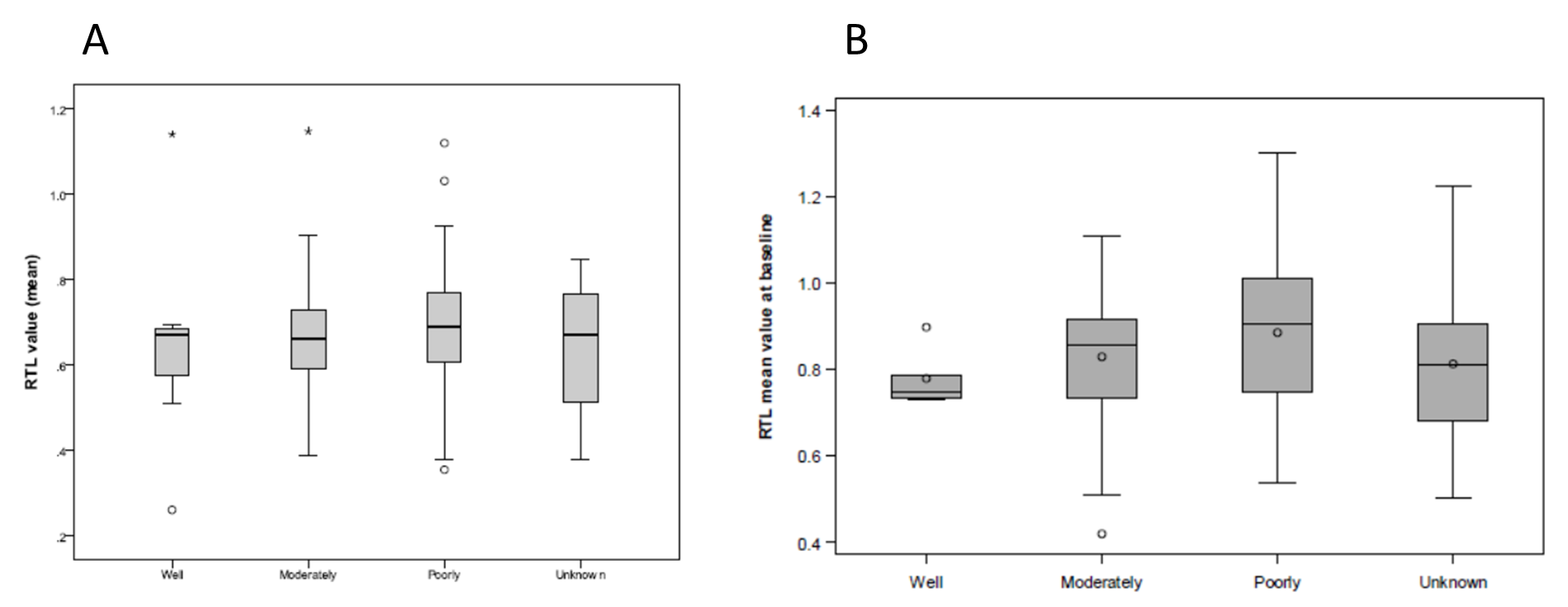
 Supporting figure S4: No significant association between RTL and primary site.** (A), advanced disease; (B), perioperative. In the perioperative study, patients with poorly differentiated tumours may have slightly longer baseline RTL in PBMCs, although this did not reach significance at the 5% level. Comparisons between baseline RTL and site were made by Kruskal-Wallis test (p = 0.492, advanced disease; p = 0.095, perioperative).

**
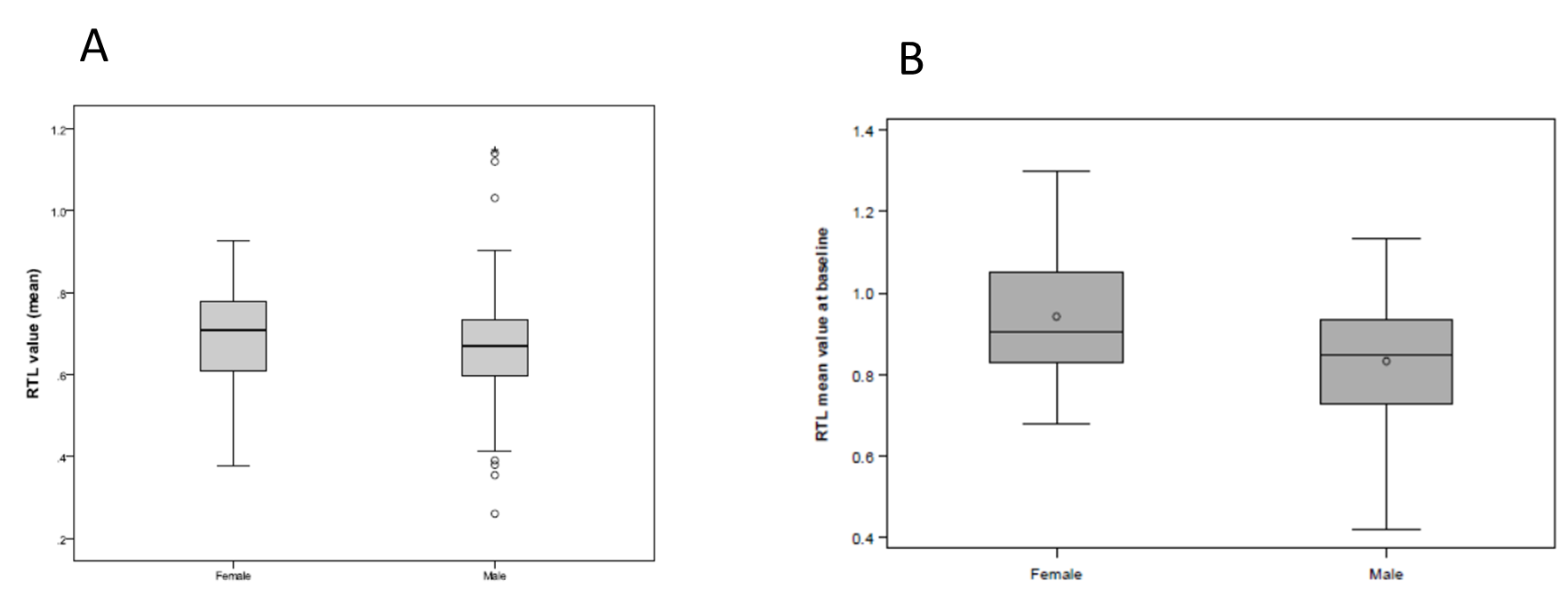
**

**Supporting figure S5: Association between RTL and female gender in perioperative study but not advanced disease.** (A), advanced disease; (B), perioperative. Comparisons were made by Mann-Whitney test. There was no significant association between baseline RTL and gender in advanced disease (p = 0.252). However, in the perioperative cohort, male patients had shorter RTL in PBMCs at baseline (p = 0.013).

**
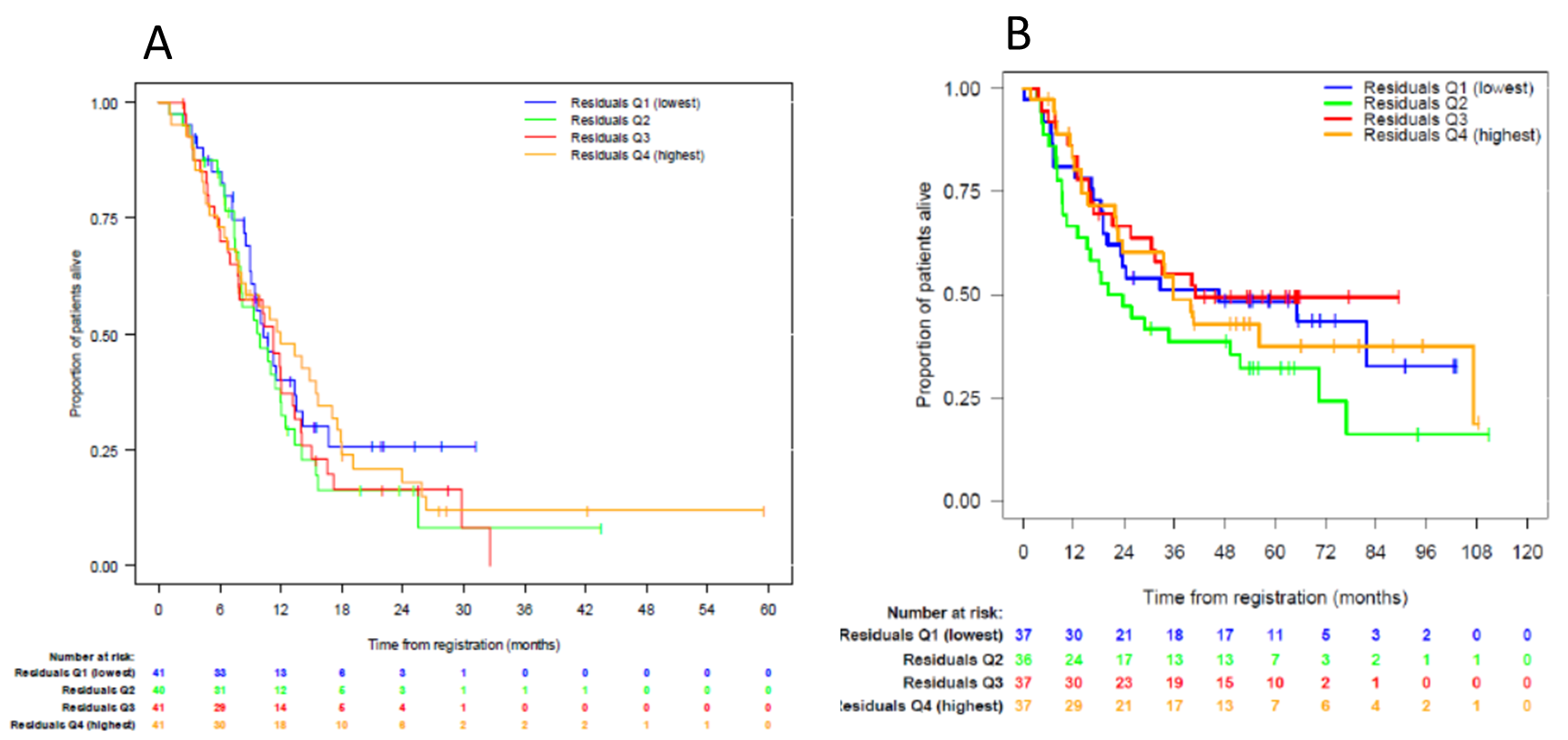
**

**Supporting figure S6: Kaplan-Meier plots of the age-adjusted and age/gender-adjusted OS models.** Since age, and age/gender were found to be significantly associated with RTL in advanced and perioperative studies, respectively, a sensitivity analysis was performed adjusting for these. Kaplan-Meier plots are for illustrative purposes only. As explained in the main text, linear models were fit for the baseline RTL data, with age and/or gender as the explanatory variables. The residuals from these models were then extracted and used as the covariates in Cox models. (A), advanced disease; (B), perioperative. The adjusted models did not suggest a strong relationship between OS and whether RTL is higher/lower than expected given age or age/gender (advanced disease: p = 0.279; perioperative: p = 0.996).

| **Endpoint (MFP transformation)** | **N (events)** | **B** | **SE** | **p-value (FDR adjusted)** | **exp B** | **90% CI for exp B** | |
| --- | --- | --- | --- | --- | --- | --- | --- |
|  |  |  |  |  |  | **lower** | **Upper** |
| **Advanced study** | | | | | | | |
| **IL12 PFS** | 182 (166) | 0.044 | 0.161 | 0.871 | 1.045 | 0.802 | 1.362 |
| **IL12 OS** | 182 (148) | 0.133 | 0.170 | 0.545 | 1.142 | 0.864 | 1.511 |
| **IL13 PFS** | 182 (166) | -0.128 | 0.157 | 0.518 | 0.880 | 0.680 | 1.138 |
| **IL13 OS** | 182 (148) | -0.054 | 0.166 | 0.744 | 0.947 | 0.721 | 1.245 |
| **IL1β PFS** | 182 (166) | 0.360 | 0.192 | 0.175 | 1.433 | 1.045 | 1.963 |
| **IL1β OS** | 182 (148) | 0.380 | 0.197 | 0.124 | 1.463 | 1.058 | 2.022 |
| **IL2 PFS** | 182 (166) | 0.392 | 0.285 | 0.317 | 1.480 | 0.927 | 2.363 |
| **IL2 OS** | 182 (148) | 0.349 | 0.283 | 0.340 | 1.418 | 0.891 | 2.257 |
| **IL4 PFS** | 182 (166) | 0.639 | 0.419 | 0.317 | 1.894 | 0.951 | 3.774 |
| **IL4 OS** | 182 (148) | 0.826 | 0.425 | 0.137 | 2.285 | 1.137 | 4.594 |
| **IFNγ/10 PFS** | 182 (166) | -0.002 | 0.012 | 0.874 | 0.998 | 0.979 | 1.018 |
| **IFNγ/10 OS** | 182 (148) | 0.008 | 0.011 | 0.554 | 1.008 | 0.990 | 1.027 |
| **IL10^-0.5 PFS** | 181 (165) | -0.346 | 0.119 | 0.020 | 0.708 | 0.582 | 0.860 |
| **IL10^-1 OS** | 181 (147) | -0.117 | 0.038 | 0.005 | 0.890 | 0.836 | 0.947 |
| **IL6/10 PFS** | 182 (166) | 0.280 | 0.134 | 0.175 | 1.323 | 1.061 | 1.649 |
| **Log(IL6/10) OS** | 182 (148) | 0.401 | 0.088 | <0.001 | 1.494 | 1.293 | 1.726 |
| **IL8/10 PFS** | 182 (166) | 0.085 | 0.022 | 0.020 | 1.088 | 1.049 | 1.129 |
| **Log(IL8/10) OS** | 182 (148) | 0.398 | 0.089 | <0.001 | 1.488 | 1.286 | 1.722 |
| **TNFα/10 PFS** | 181* (165) | 0.368 | 0.395 | 0.516 | 1.445 | 0.754 | 2.768 |
| **TNFα/10 OS** | 181* (147) | 0.889 | 0.418 | 0.105 | 2.433 | 1.223 | 4.837 |

**Supporting table S1: Summary of Cox regression results for PFS/OS using the method of fractional polynomials for all cytokines in the advanced study.** IL1β**,** IL2, IL4, IL12, an IL13 were treated as categorical assays (detected/not detected) and MFP was not performed. * For TNFα, one outlier patient was excluded. When Cox regression was performed with this patient for PFS, we found HR = 1.377, 90% CI = 1.199 – 1.582, unadjusted p = 0.006. After exclusion, unadjusted p = 0.361, with the other results reported above. For the OS model, with the inclusion of this patient, we obtained HR = 1.332, 90% CI = 1.182 – 1.501, unadjusted p = 0.006. After exclusion, unadjusted p = 0.042, with the other results reported above. Since these results depend so heavily on a single patient, and since the OS univariate model without this patient did not remain significant after adjustment for FDR, TNFα was not considered further. For IL10 analysis, one patient was excluded due to missing IL10 data.
